# On the Strain Rate Sensitivity of Fused Filament Fabrication (FFF) Processed PLA, ABS, PETG, PA6, and PP Thermoplastic Polymers

**DOI:** 10.3390/polym12122924

**Published:** 2020-12-06

**Authors:** Nectarios Vidakis, Markos Petousis, Emmanouil Velidakis, Marco Liebscher, Viktor Mechtcherine, Lazaros Tzounis

**Affiliations:** 1 Mechanical Engineering Department, Hellenic Mediterranean University, 71410 Heraklion, Greece; vidakis@hmu.gr (N.V.); mvelidakis@hmu.gr (E.V.); 2 Institute of Construction Materials, Technische Universität Dresden, DE-01062 Dresden, Germany; viktor.mechtcherine@tu-dresden.de; 3 Department of Materials Science and Engineering, University of Ioannina, 45110 Ioannina, Greece; latzounis@uoi.gr

**Keywords:** additive manufacturing (AM), three-dimensional (3D) printing, fused filament fabrication (FFF), strain rate sensitivity, tensile properties, polylactic acid (PLA), acrylonitrile-butadiene-styrene (ABS), polyethylene terephthalate glycol (PETG), polyamide 6 (PA6), polypropylene (PP)

## Abstract

In this study, the strain rate sensitivity of five different thermoplastic polymers processed via Fused Filament Fabrication (FFF) Additive Manufacturing (AM) is reported. Namely, Polylactic Acid (PLA), Acrylonitrile-Butadiene-Styrene (ABS), Polyethylene Terephthalate Glycol (PETG), Polyamide 6 (PA6), and Polypropylene (PP) were thoroughly investigated under static tensile loading conditions at different strain rates. Strain rates have been selected representing the most common applications of polymeric materials manufactured by Three-Dimensional (3D) Printing. Each polymer was exposed to five different strain rates in order to elucidate the dependency and sensitivity of the tensile properties, i.e., stiffness, strength, and toughness on the applied strain rate. Scanning Electron Microscopy (SEM) was employed to investigate the 3D printed samples’ fractured surfaces, as a means to derive important information regarding the fracture process, the type of fracture (brittle or ductile), as well as correlate the fractured surface characteristics with the mechanical response under certain strain rate conditions. An Expectation–Maximization (EM) analysis was carried out. Finally, a comparison is presented calculating the strain rate sensitivity index “m” and toughness of all materials at the different applied strain rates.

## 1. Introduction

Additive Manufacturing (AM) has recently gained a large attention from the industry as well as the academic community, while it is considered to be in the forefront of industrial manufacturing processes either for prototyping or mass production procedures. Especially Fused Filament Fabrication (FFF) technology, which has been mainly used for prototyping purposes until now, opens new avenues expanding its capabilities to small-batched mass production [1]. As reported from recent studies, a raise of over 400% in revenue of the AM market is expected for 2020 in comparison to 2016 [2]. This is expected to lead to a vastly increasing need for high-performance engineering thermoplastic materials in FFF technology. Industrialization of the method requires a necessarily good knowledge of the mechanical properties of polymers used for end-use products manufactured by FFF.

The strain rate sensitivity of polymers has been extensively studied in the literature [3,4,5,6,7]. When strain rate sensitivity comes to AM processed polymeric materials, only few studies have been reported [4]. AM technologies and especially FFF provide the ability to create parts with more complex geometries than the traditional manufacturing methods, i.e., injection molding, casting from polymer solutions, etc. AM technologies are creating parts using a “layer by layer” principle, which finally endows an anisotropic behavior [8]. This creates a need of implementing focused studies on the 3D printed specimens printing parameters and their effect on the mechanical response of the polymers. Song et al. reported different properties of polymers processed via conventional methods other than AM technologies [9]. Namely, it has been reported that 3D printed parts exhibit increased crystallinity, increased fracture toughness, and increased sensitivity to the strain rate.

The effect of the 3D printing parameters on the mechanical properties of the resulting 3D printed component has been widely reported in the literature [10,11,12]. Layer thickness, infill orientation, and number of shell perimeters have been tested for the PLA polymer by Lanzotti et al. [13]. Hibbert et al. conducted a similar experiment regarding the ABS polymer [14]. Johnson and French [15] have also reported results for the infill effect on 3D printed PETG and PA6 specimens’ mechanical properties. Carneiro et al. investigated similar parameters for 3D printed PP specimens [16].

The propagation of defect is highly dependent on the fracture toughness of the material, with the fracture toughness to the strain rate effect being reported several times and experimentally tested in the literature. Some interesting results have been conducted to an experimental research of Vu-Khanh and Fisa [17]. In this study, PP reinforced with glass flakes has shown a decreasing rate on fracture toughness, while increased strain rates were implemented. Todo et al. reported on reinforced polyamide 6 composites under various strain rate conditions [18]. In this study, results are also revealing an increasing rate on the calculated tensile strength, with the increase of the strain rate. Cantwell and Blyton [19] reported a low dependency of the fracture toughness to the strain rate. Specifically, a carbon fiber Reinforced Polymer (CFRP) composite using an epoxy matrix, as well as glass fiber Reinforced Polymer (GFRP) composite of a PP thermoplastic matrix were investigated.

In this work, in order to test the strain rate sensitivity of Polylactic Acid (PLA), Acrylonitrile-Butadiene-Styrene (ABS), Polyethylene Terephthalate Glycol (PETG), Polyamide 6 (PA6), and Polypropylene (PP) polymers and establish a comparable evaluation approach in the study, most of the FFF 3D printing parameters have been kept identical for all polymers. Regarding the strain rate sensitivity, it was experimentally determined and reported in the literature, that the strain rate highly affects the tensile properties [20,21,22,23]. Wang et al. [24] conducted experimental tests on the strain rate sensitivity of a polyamide 6-based composite. In this study, the strain rate sensitivity is reported on different structural geometries. As the strain rate increases, the infill’s geometry highly affects the composites behavior. The anisotropy of additively manufactured structures creates a need for a deep investigation of the strain rate sensitivity.

Modifications on the mechanical properties due to the effect of the strain rate on polymers should be further investigated and probably modeled. In this way, designers can achieve a better understanding on the mechanical properties’ specifications on each case study. A possible approach to achieve better mechanical properties on structures created through AM can be conducted using the strain rate sensitivity as a design factor.

In the study at hand, the strain rate sensitivity under quasi-static tensile loading conditions of five different 3D printed thermoplastic polymers is reported. Thermoplastic materials widely used in AM and especially in FFF 3D printing technology have been experimentally investigated. Specimens were tested in five different tensile speeds and results were analyzed and evaluated. A study is conducted by calculating the strain rate sensitivity index “m” and the toughness at different strain rates for each one of the polymeric material studied. In all cases, the yield strength and the tensile strength slightly increase with the increase of the strain rate, as expected, due to the strain hardening phenomena. However, interestingly enough, it has been found that the strain rate sensitivity varies both as a function of the different thermoplastic polymers tested, as well as with respect to the applied strain rate.

## 2. Materials and Methods

### 2.1. Materials

Five different polymers have been selected for testing. The Polylactic Acid (PLA) polymer in coarse powder was procured from Plastika Kritis S.A. (Heraklion, Crete, Greece) of 3052D grade. The ABS Terluran HI 10 polymer was procured from INEOS Styrolution in a powder form. Additionally, PETG procured from Felfil in pellet form, PA6 Novamid N-X 160 procured from DSM Engineering Materials in pellet form, and KRITILEN PP procured from Plastika Kritis S.A. in coarse powder form have been used for this experimental study. All polymers are well known materials in the AM industry, and are widely used in 3D printing, especially in the FFF process.

### 2.2. Methods

**Figure 1:**
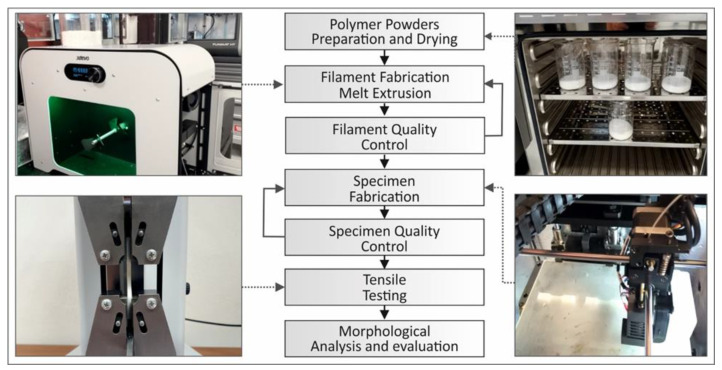
An overview diagram demonstrating the whole process followed to manufacture and test the five different polymeric samples.

Figure 1 shows a diagram of the process followed to manufacture and test the different polymeric material samples. Each step of this process is described in detail in the following sub-sections.

#### 2.2.1. Extrusion Procedure

All polymers used in this research were extruded to a 1.75 mm diameter filament using a 3D Evo Composer 450 desktop extruder. As expected, different extruding conditions are required and they were used for each polymer. Preliminary work was done for the determination of these extrusion’s specifications for each polymer. An experimentation procedure was followed, to achieve the best filament quality for each polymer. The manufacturer’s recommendations over temperatures and other extrusion parameters have been considered during the extrusion process. The extrusion’s specifications for each polymer can be seen in Table 1. Before the extrusion procedure, each polymer was dried for 12 h at 80 °C (for PLA the temperature has been set to 50 °C).

#### 2.2.2. Tensile Specimens’ Fabrication

The specimens for the tensile tests were manufactured according to the American Society for Testing and Materials (ASTM) D638-02a standard (type V specimens with 3.2 mm thickness). In total, 125 specimens were 3D printed consisting of 25 specimens for each polymer scenario. Specimens were fabricated with the Fused Filament Fabrication (FFF) Additive Manufacturing technology, using an Intamsys Funmat HT 3D Printer (Intamsys Technology Co. Ltd., Shanghai, China). All specimens have been produced using the same 3D printing settings regarding the shell pattern (a shell with two perimeters was set) and the infill.

The infill was set to 100% and the deposition pattern direction was set to 45/135° alternating at each layer. All specimens were produced using the same orientation, with the longest side of the specimen on the XY plane of the 3D printer. The 3D printer’s nozzle was common for all materials and has a diameter of 0.4 mm. The default settings of the Intamsuite (slicer software of Intamsys HT) software were employed for the flow rate, retraction, and other settings regarding the material flow.

Same with the extrusion process, a preliminary experimental process was followed, for the determination of specific 3D printing settings, which are required for each polymer, in order to be able to be 3D printed. Table 2 shows the 3D printing settings that differ for each polymer.

#### 2.2.3. Testing Procedure

The tensile tests were performed using an Imada MX2 (IMADA, IL, USA) tension test apparatus, using standardized grips. A range of 10 to 100 mm/min in tension speed has been selected in order to evaluate the performance of each polymer under the different test speeds. Five specimens for each speed scenario have been tested for each polymer at room temperature (room temperature: 23 to 25 °C). The tested speeds were 10, 25, 50, 75, and 100 mm/min. These speeds were selected, as they cover a wide range of the strain rates polymers support in their typical working conditions, so the experimental data on 3D printed specimens at this strain rate range are considered to be more useful for future usage as design parameters. Additionally, the corresponding tensile standard (ASTM D638) suggests for this specific specimen (type V) to use either 10 or 100 mm/min as the speed of testing, with 10 mm/min being the most common loading speed in industrial applications. Hence, the specimens were selected to be tested in a speed range within the range of the standard, with specimens tested in five different speeds, to determine the effect of the strain rate in the mechanical response of the polymers tested.

#### 2.2.4. Characterization Techniques

The FEI NanoSem 200 (FEI, Eindhoven, The Netherlands) operating at an accelerating voltage of 1–2 kV was utilized in order to study the 3D printed samples’ side surface as well as the fractured surfaces of the different polymers at the lower and the higher strain rate of the study, namely 10 and 100 mm/min, respectively. A thin layer (3 nm) of platinum was deposited by sputtering onto all the samples that have been investigated by SEM in this study in order to avoid charging effects, as reported elsewhere [25]. 

## 3. Results

### 3.1. PLA: Tensile Properties as a Function of the Strain Rate

**Figure 2:**
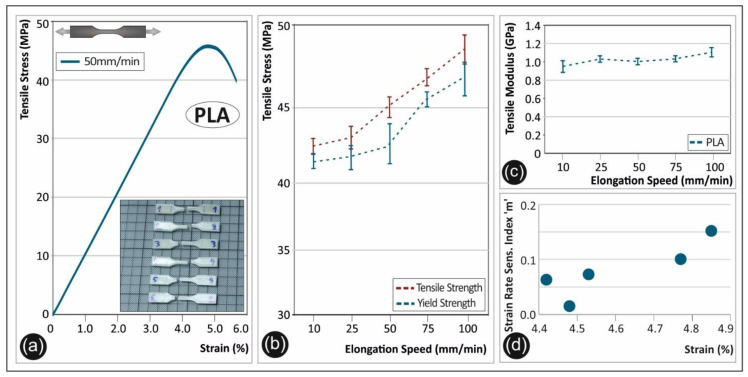
(**a**) Representative tensile stress (MPa)—strain (%) curve of polylactic acid (PLA) at a 50 mm/min elongation speed, (**b**) average tensile strength and yield strength (with the corresponding standard deviation) for PLA in all five elongation speeds, (**c**) tensile modulus (with the corresponding standard deviation) of PLA under the five different elongation speeds, and (**d**) strain rate sensitivity index “m”—strain (%) graph for PLA.

In Figure 2, the experimental results of the PLA polymer tensile test are shown. A representative stress-strain curve is shown in Figure 2a, from one of the specimens tested at 50 mm/min. The rather brittle behavior of PLA could be clearly observed. A difference in the tensile strength and the yield strength occurs for the different elongation speeds, as shown in Figure 2b. Namely, an increase of 16.1% was found for the maximum tensile strength at a strain rate of 100 mm/min in comparison to the lowest strain rate of 10 mm/min. Moreover, the tensile modulus plotted in Figure 2c shows a similar trend with the maximum tensile strength with the increased strain rate. Specifically, the same increase was found for the elongation speed of 100 mm/min when compared to 10 mm/min. The increase in the maximum tensile strength, yield strength, and tensile modulus of elasticity seems to be rather more intense for elongation speeds higher than 50 mm/min, as shown in Figure 2b,c, respectively. Figure 2d represents a graph of the calculated strain rate sensitivity index “*m*” with respect to the corresponding strain values (%) for each case. Index “*m*” was calculated by the following Equation (1) [4,7]:

(1) $$m=\frac{\Delta ln\left(\sigma \right)}{\Delta ln(\dot{\varepsilon )}}$$

The strain rate sensitivity “*m*” factor is a parameter related to the ability of a material to withstand static mechanical loads of different strain rates. The graphs shown in Figure 2 and especially the calculation of the “*m*” index (Figure 2d), show not only the brittleness of PLA, but also elaborate the polymer’s strain rate limit.

### 3.2. ABS: Tensile Properties as a Function of the Strain Rate

**Figure 3:**
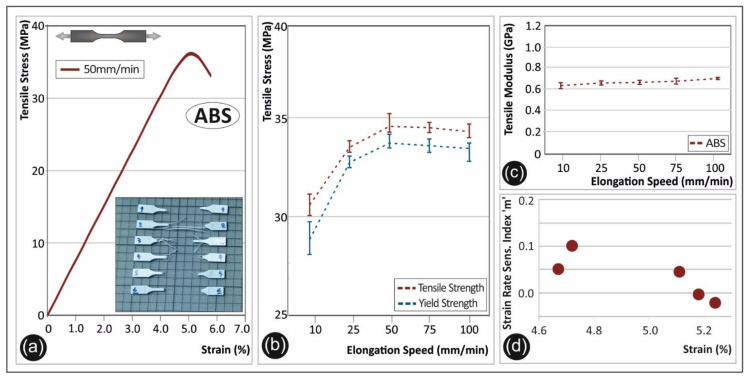
(**a**) Representative tensile stress (MPa)—strain (%) curve of acrylonitrile-butadiene-styrene (ABS) at a 50 mm/min elongation speed, (**b**) average tensile strength and yield strength (with the corresponding standard deviation) for ABS in all five elongation speeds, (**c**) tensile modulus (with the corresponding standard deviation) of ABS under the five different elongation speeds, and (**d**) strain rate sensitivity index “m”—strain (%) graph for ABS.

Figure 3 shows the corresponding tensile tests and the derived mechanical properties of the ABS polymer. A representative stress-strain curve is shown in Figure 3a, from one of the specimens tested at 50 mm/min. In Figure 3b, it can be observed that the maximum tensile strength and yield strength have a steep increase when the elongation speed increases from 10 to 25 mm/min. In this case, a 13.7% increase of yield strength was observed. The tensile modulus plotted in Figure 3c is slightly changing for the different applied strain rates. An average increase of 6.0% was observed from the lowest elongation speed to the highest one.

ABS is a well-known material used in different applications due to its plastic yield at high strain rates accompanied with toughness and an underlying ductile fracture mechanism [10]. Therefore, ABS is used to withstand high strain rate loads in different engineering applications. The calculated strain rate sensitivity index “m”, which is shown in Figure 3d confirms this behavior of ABS. Low “m” index values reveal a rather non-dependent behavior to the rate that the load is applied, corroborating the results from a previous study [7]. Even at high strain rates of mechanical loading, ABS exhibits a similar mechanical response.

### 3.3. PETG: Tensile Properties as a Function of the Strain Rate

**Figure 4:**
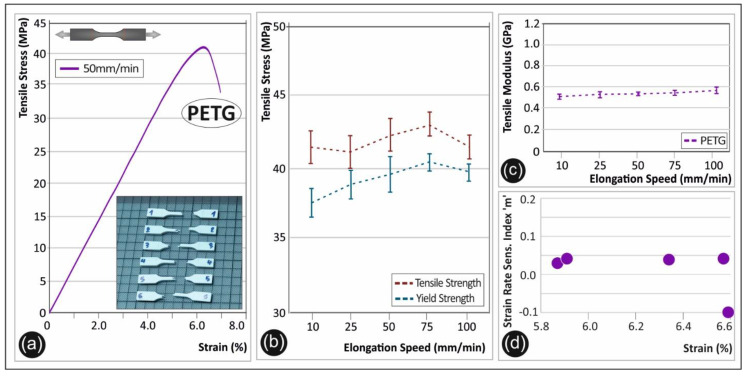
(**a**) Representative tensile stress (MPa)—strain (%) curve of polyethylene terephthalate glycol (PETG) at a 50 mm/min elongation speed, (**b**) average tensile strength and yield strength (with the corresponding standard deviation) for PETG in all five elongation speeds, (**c**) tensile modulus (with the corresponding standard deviation) of PETG under the five different elongation speeds, and (**d**) strain rate sensitivity index “m”—strain (%) graph for PETG.

In Figure 4, the experimental results of the PETG polymer tensile test are shown. A representative stress-strain curve is shown in Figure 4a, from one of the specimens tested at 50 mm/min. PETG is a well-known material used in additive manufacturing and especially in FFF based technology 3D printers. The material’s strain rate sensitivity was found to be rather low. A slight increase of 8.5% for the maximum tensile stress was observed from the lowest elongation speed to 75 mm/min (Figure 4b). A similar increase occurred also for the yield strength. The overall increase of the yield strength from the lowest to the highest elongation speed is ~5.5%. The average tensile modulus of elasticity as a function of the elongation speed is shown in Figure 4c, demonstrating a rather constant behavior throughout all the applied strain rates. The strain rate sensitivity of PETG seems to have a low sensitivity index “m” value (< 0.1). The calculated “m” index values are rather stable for all strains developed on the polymer, as illustrated in Figure 4d.

### 3.4. PA6: Tensile Properties as a Function of the Strain Rate

**Figure 5:**
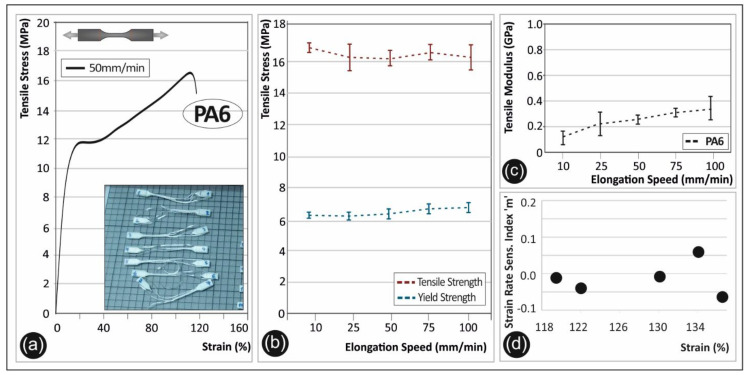
(**a**) Representative tensile stress (MPa)—strain (%) curve of polyamide 6 (PA6) at a 50 mm/min elongation speed, (**b**) average tensile strength and yield strength (with the corresponding standard deviation) for PA6 in all five elongation speeds, (**c**) tensile modulus (with the corresponding standard deviation) of PA6 under the five different elongation speeds, and (**d**) strain rate sensitivity index “m”—strain (%) graph for PA6.

In Figure 5, the experimental results of the PA6 polymer tensile test are shown. PA6 exhibited a relatively weak elastic region with an intense plastic deformation already starting from low stress levels. A representative stress-strain curve is shown in Figure 5a, from one of the specimens tested at 50 mm/min. A stress at break of ~16.2 MPa was observed for the elongation speed of 50 mm/min. The tensile properties of PA6 obtained in this work are similar to other AM manufactured PA6 specimens reported elsewhere [26]. In Figure 5b, a slight decrease of 4.1% is observed at an elongation speed of 50 mm/min for the maximum tensile stress. PA6 seems to have a rather stable yield strength value as a function of the different strain rates. A slight increase of 7% is found regarding the yield strength (Figure 5b). The tensile modulus of elasticity is increasing about 21.5% from the lowest to the highest elongation speed (Figure 5c). The calculated strain rate sensitivity “m” index values are found to be around zero (Figure 5d). It is worth mentioning that PA6 exhibited the lowest “m” values amongst the five polymers investigated in this study.

### 3.5. PP: Tensile Properties as a Function of the Strain Rate

**Figure 6:**
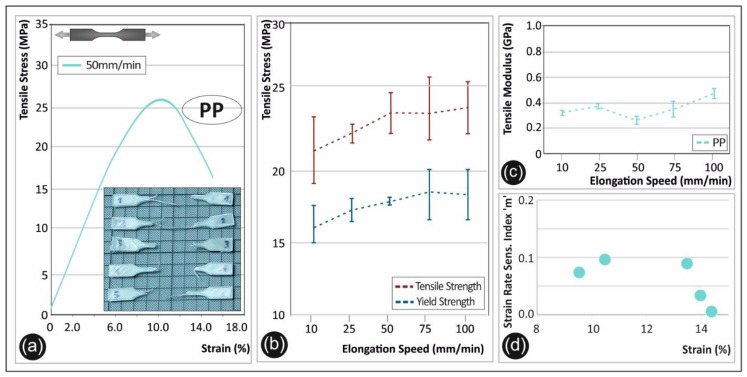
(**a**) Representative tensile stress (MPa)—strain (%) curve of polypropylene (PP) at a 50 mm/min elongation speed, (**b**) average tensile strength and yield strength (with the corresponding standard deviation) for PP in all five elongation speeds, (**c**) tensile modulus (with the corresponding standard deviation) of PP under the five different elongation speeds, and (**d**) strain rate sensitivity index “m”—strain (%) graph for PP.

Figure 6 summarizes the tensile test results of the PP polymer. A representative stress-strain curve is shown in Figure 6a, from one of the specimens tested at 50 mm/min. In general, an increase in the maximum strength could be observed when the strain rate increases (Figure 6b). Specifically, for the 25 mm/min elongation speed, an increase of >16% is found as compared to the 10 mm/min; while a further increase of the same magnitude could be observed by increasing further the elongation speed to 50 mm/min. In Figure 6b, the average values of the maximum tensile strength and the yield strength of PP are shown for the five different elongation speeds. Both the maximum tensile strength and the yield strength are increasing. The tensile strength is calculated to have a total increase of ~27% from the lowest elongation speed of 10 mm/min to the highest one of 100 mm/min. A similar trend of increase was observed also for the yield strength. The tensile modulus of elasticity shown in Figure 6c is reported to have a total increase of approximately 23% from lowest to highest strain rates. In Figure 6d, the calculated “m” sensitivity index for PP is presented. PP appears to have relatively low “m” strain rate sensitivity values.

### 3.6. SEM Morphological Characterization

**Figure 7:**
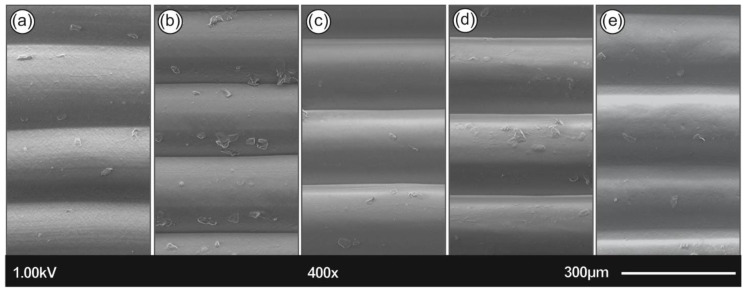
Side focus on 3D printed specimens of all five polymers tested; namely (**a**) PLA, (**b**) ABS, (**c**) PETG, (**d**) PA6, and (**e**) PP.

Figure 7 summarizes the SEM images of the 3D printed polymers, namely, PLA (Figure 7a), ABS (Figure 7b), PETG (Figure 7c), PA6 (Figure 7d), and PP (Figure 7e). All images were acquired from 3D printed samples fabricated with a 100 µm printed layer thickness.

### 3.7. Fractography Microstructure Investigations

**Figure 8:**
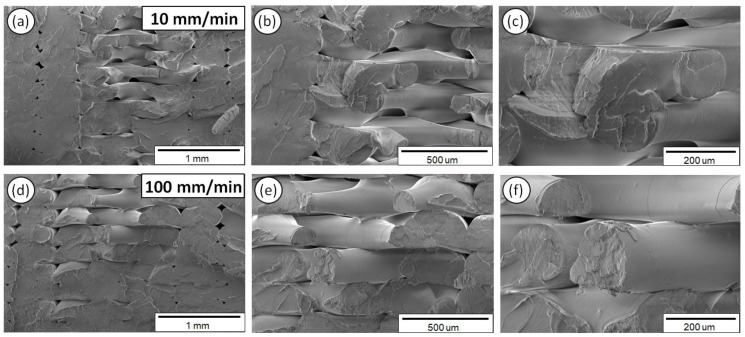
SEM images of PLA fractured surfaces at three different magnifications for the 10 (**a**–**c**) and 100 mm/min (**d**–**f**) strain rates.

Figure 8 demonstrates the PLA fractured surface microstructural characteristics after the tensile tests performed at 10 (Figure 8a–c) and 100 mm/min (Figure 8d–f) strain rates, at three different magnifications in order to elaborate the underlying macroscopic, as well as micron-scale failure mechanisms, i.e., brittle or ductile fracture mechanism.

**Figure 9:**
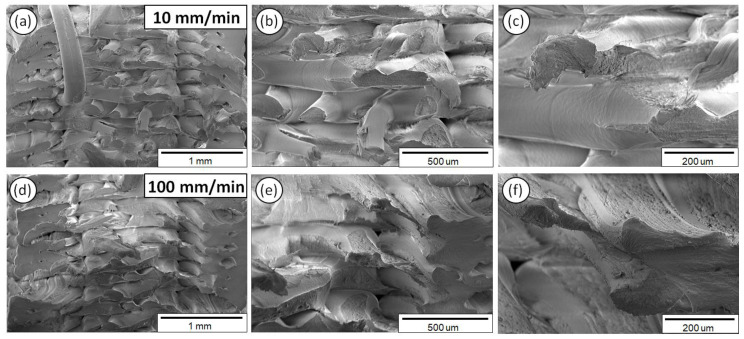
SEM images of ABS fractured surfaces at three different magnifications for the 10 (**a**–**c**) and 100 mm/min (**d**–**f**) strain rates.

Figure 9 illustrates accordingly the fractured surfaces of 3D printed ABS samples exposed to 10 (Figure 9a–c) and 100 mm/min (Figure 9d–f) strain rates, at three different magnifications.

**Figure 10:**
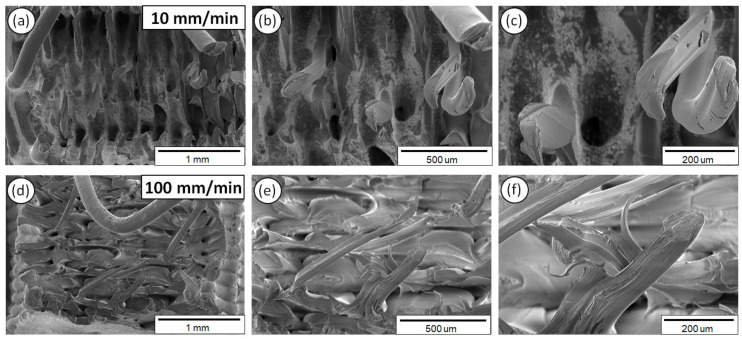
SEM images of PETG fractured surfaces at three different magnifications for the 10 (**a**–**c**) and 100 mm/min (**d**–**f**) strain rates.

Figure 10 illustrates the fractured surface characteristics of 3D printed PETG samples exposed to 10 (Figure 10a–c) and 100 mm/min (Figure 10d–f) strain rates, at three different magnifications.

**Figure 11:**
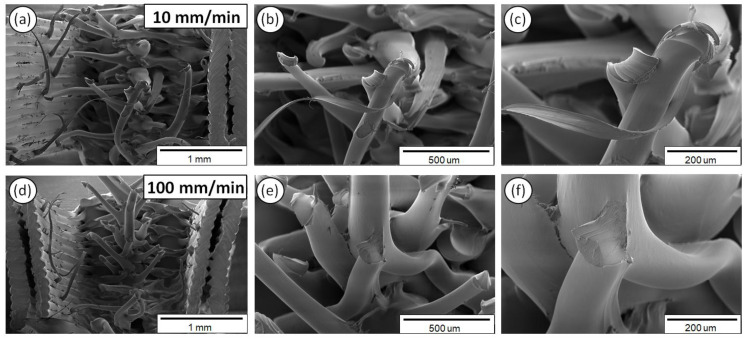
SEM images of PA6 fractured surfaces at three different magnifications for the 10 (**a–c**) and 100 mm/min (**d–f**) strain rates.

Figure 11 shows the PA6 fractured surface characteristics exposed to 10 (Figure 11a–c) and 100 mm/min (Figure 11d–f) strain rates, at three different magnifications.

**Figure 12:**
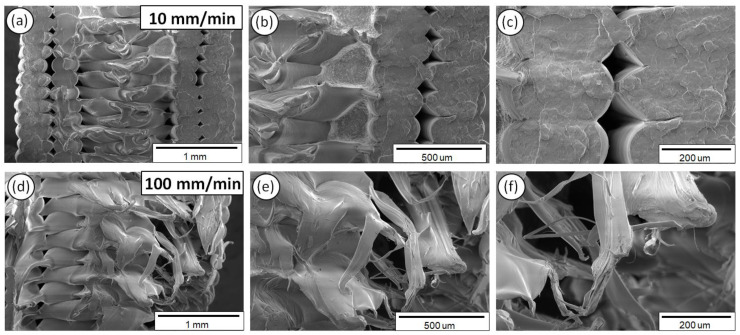
SEM images of PP fractured surfaces at three different magnifications for the 10 (**a**–**c**) and 100 mm/min (**d**–**f**) strain rates.

Finally, in Figure 12, the fractured surfaces of 3D printed PP samples are depicted tested at 10 (Figure 12a–c) and 100 mm/min (Figure 12d–f) strain rates, shown at three different magnifications.

## 4. Discussion

The strain rate effect on the tensile strength, yield strength, and elastic modulus is thoroughly studied in this work for five different thermoplastic polymers extensively used in AM nowadays. The results clearly show the strain rate sensitivity behavior of the specimens manufactured by the 3D printing FFF technology herein (Figure 2, Figure 3, Figure 4, Figure 5 and Figure 6 tensile test results).

In Figure 7, the SEM images of the 3D printed samples’ side surface corroborate the 3D printing layer thickness characteristics; being in a good agreement with the resolution of the Intamsys Funmat HT 3D printer manufacturer’s technical specifications in terms of 3D printer’s resolution. In addition, all samples exhibit a homogeneous 3D printed layer thickness without any observed micronized voids and/or discontinuities in the interlayers attributed to the optimum 3D printing parameters used in the study. From the SEM images, it can be further deduced that high quality 3D printed objects have been manufactured with good adhesion between the layers, which could most likely result in the high mechanical performance of the 3D printed components.

Polymers are known to be viscoelastic materials, while their mechanical behavior highly depends on the rate that an external mechanical load is applied, as well as the external environment temperature, otherwise defined as operational temperature. Therefore, upon designing and further manufacturing a polymer-based part, it is of utmost importance to study and specify the effect of, i.e., strain rate or external temperature that the part will be exposed to, since they can significantly affect the part’s mechanical performance in the operational environment. Namely, all the tensile test experiments were performed at a constant and controlled temperature in this study (room temperature: 23 to 25 °C), while varying only the strain rate levels.

As it can be observed from the SEM fractography investigations (Figure 8, Figure 9, Figure 10, Figure 11 and Figure 12), each polymeric material shows a specific fracture mechanism due to the inherent stiffness, while only in the case of PLA, ABS, PETG, and PP the fracture mechanism was found to be dependent on the applied strain rate used for the static tensile test experiments. The strain rate is the reciprocal of time. Longer times imply lower strain rates, while shorter times are related to higher strain rates. As such, in the SEM images for all samples shown at two representative strain rates, namely low (10 mm/min) and high strain rate (100 mm/min), a relatively brittle fracture mechanism could be observed for the low strain rates, whereas a ductile fracture mechanism for the high strain rate due to the strain hardening developing phenomena. In the case of PA6, it is known and it has been demonstrated also by the corresponding stress-strain curve at 50 mm/min (Figure 5a) that as a polymeric material, it has relatively low stiffness, especially compared to the other thermoplastic materials investigated in this study, while it undergoes a prominent plastic deformation that results into a ductile fracture mechanism.

For the low strain rates, no strain hardening phenomena are expected so that polymeric chains could obtain, for instance, some orientation resulting further in a tougher behavior, increasing the materials’ resistance to deformation and making the material capable of carrying a higher amount of load. Therefore, a rather brittle fracture mechanism could be seen for the low strain rates with relatively smooth fractured surfaces, apart from the case of PA6. As previously mentioned, PA6 has inherently a very pronounced plastic regime, while not being affected by the applied strain rates towards any possible development of a strain hardening mechanism. For the low strain rates, PLA, ABS, PETG, and PP materials show less resistance to deformation, while during the fracture and at the point that the sample cannot take up more load, the sample fails within its elastic regime/partially having entered the plastic regime, while the energy that has been stored to the macromolecular chains is abruptly released, thus resulting into relatively smoother fractured surfaces (no observed pulled-out fibers, ductile phenomena, etc.).

On the other hand, a ductile fracture mechanism is expected to occur under the high strain rates due to strain hardening. Specifically, the fractured surfaces exhibited in general higher roughness and in some cases a great number of filaments have been pulled out from the sample’s surface. Moreover, the different in nature polymers could exhibit a different strain hardening effect, making them tougher and exhibiting a ductile failure. This can be directly correlated also with the strain sensitivity index “m” calculated in this study for all polymers tested at the five different strain rates. Relatively, a ductile fracture mechanism (high strain rate—100 mm/min) can be more easily observed in the case of PETG (Figure 10) and PP (Figure 12) fractured surfaces, while only slightly higher roughness and pulled-out fibers could be observed for the PLA and ABS fractured surfaces at 100 mm/min (compared to the 10 mm/min low strain rate fractured surfaces).

The toughness calculation (derived by the calculation of the area under the corresponding stress-strain curve in all cases) is crucial regarding applications that require high strain rate mechanical loads. The strain rate sensitivity index “m” is also an important measure in order to proceed with a right choice of polymer material choice. Even if polymers are usually used for low loading applications, the rate of the load applied may even drive to “fatal fracture”.

**Figure 13:**
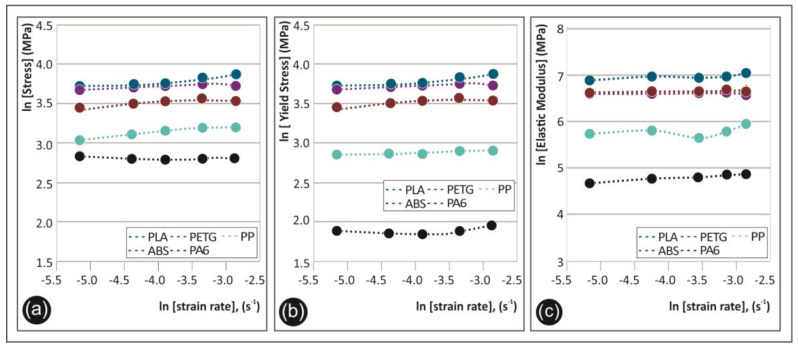
ln(x) to ln (strain rate), (s^−1^) for all five polymers tested, where x = (**a**) maximum tensile stress (MPa), (**b**) yield stress (MPa), and (**c**) tensile modulus of elasticity (MPa).

The importance of Figure 13 relies on demonstrating the ability of each 3D printed thermoplastic polymeric material in this study to withstand static mechanical loads in the tensile mode under different strain rate loading conditions. More specifically, the plots of the maximum tensile stress (Figure 13a), yield stress (Figure 13b), and tensile modulus (Figure 13c) for all the five different strain rates (s^−1^) in ln scale are shown. Figure 13 is expressing the strain rate sensitivity index “m” in a more comparable way for all five polymers tested, since it shows the trend for each mechanical property with the increase of the strain rate.

**Figure 14:**
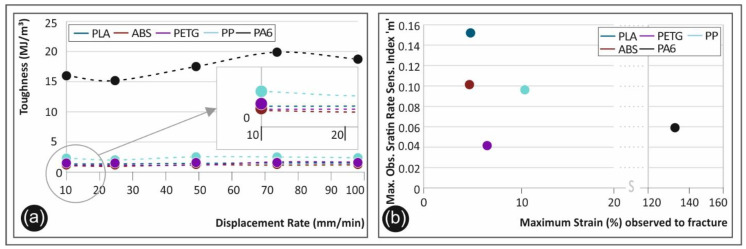
(**a**) Toughness (MJ/m^3^) to the displacement rate (mm/min) for all five tested polymers, and (**b**) maximum strain rate sensitivity index “m” calculated to the corresponding strain (%) of the specimen for all five polymers tested.

Figure 14a shows the calculated toughness for each material for all five elongation speeds tested, whereby Figure 14b shows the maximum calculated sensitivity index “m” to the corresponding maximum strain (%) at the fracture. From Figure 14a, it can be deduced that PA6 is a material that can be used in applications where the “steep” loads exists. All other polymers, namely PLA, ABS, PETG, and PP have the potential to be further investigated during the design procedure. In Figure 14b, it can be seen that PLA seems to be a rather high dependent material to the strain rate (high strain rate sensitivity). ABS and PP seem to have a similar behavior on the strain rate effect. PETG and PA6 are shown to have a rather low dependence to the strain rate effect as index “m” values are low (even at high strain rate values).

## 5. Conclusions

In this study, the strain rate sensitivity of five different thermoplastic polymeric materials widely used in AM FFF technology has been thoroughly studied. The 3D printing FFF AM parameters were set to the “highest quality”, i.e., 100% infill, aiming to overcome the anisotropic behavior of AM manufactured specimens. The calculations of the strain rate sensitivity index “m” and toughness for ABS, PA6, PETG, PLA, and PP can more precisely demonstrate the expected mechanical properties from each of the polymers under different static tensile loading conditions. PA6, ABS, and PP 3D printed samples were found to exhibit a low strain rate sensitivity dependency of their mechanical properties, i.e., stiffness, yield strength, tensile strength, and toughness, allowing them to be applied in various cases where low and/or high strain rates may be present. On the other hand, PLA seems to be highly sensitive to the strain rate. Relatively low strain rates with high values to the strain rate sensitivity index “m” and low toughness reveal that PLA needs to be appropriately investigated further in order to improve its mechanical properties, e.g., by adding some nucleation agents to increase its crystallinity, as for instance, some nano-scale fillers, incorporating some short fibers as reinforcement, etc. Finally, PETG showed the most promising behavior for 3D printing via FFF AM processing. PETG exhibited a low strain rate sensitivity, while taking into account the material’s “ease of processing” in 3D FFF printing. In addition, PETG could be considered as a thermoplastic material that can be widely used for 3D printing engineering applications, even if low and high strain rates/loading conditions will be applied.

## Figures and Tables

**Table 1:** Extrusion specifications used for the filament fabrication on the three-dimensional (3D) Evo Composer 450 filament extruder.

	PLA	ABS	PETG	PA6	PP
**Extruder Heat Zone 1**	170 °C	190 °C	180 °C	190 °C	195 °C
**Extruder Heat Zone 2**	190 °C	230 °C	200 °C	220 °C	205 °C
**Extruder Heat Zone 3**	190 °C	240 °C	200 °C	220 °C	205 °C
**Extruder Heat Zone 4**	170 °C	220 °C	180 °C	190 °C	195 °C
**Screw Rotation Speed (rpm)**	5	5	5	3.5	3

**Table 2:** 3D printing settings used on the Intamsys Funmat HT 3D printer for the manufacturing of the specimens.

	PLA	ABS	PETG	PA6	PP
**Print Speed (mm/s)**	40	40	40	40	30
H**otend Temperature (°C)**	215	260	240	235	230
**Bed Temperature (°C)**	60	80	70	60	105
